# Mathematical modeling of the SARS-CoV-2 epidemic in Qatar and its impact on the national response to COVID-19

**DOI:** 10.7189/jogh.11.05005

**Published:** 2021-01-16

**Authors:** Houssein H Ayoub, Hiam Chemaitelly, Shaheen Seedat, Monia Makhoul, Zaina Al Kanaani, Abdullatif Al Khal, Einas Al Kuwari, Adeel A Butt, Peter Coyle, Andrew Jeremijenko, Anvar Hassan Kaleeckal, Ali Nizar Latif, Riyazuddin Mohammad Shaik, Hanan Abdul Rahim, Hadi M Yassine, Mohamed G Al Kuwari, Hamad Eid Al Romaihi, Mohamed H Al-Thani, Roberto Bertollini, Laith J Abu Raddad

**Affiliations:** 1 Department of Mathematics, Statistics, and Physics, Qatar University, Doha, Qatar; 2 Infectious Disease Epidemiology Group, Weill Cornell Medicine-Qatar, Cornell University, Doha, Qatar; 3 World Health Organization Collaborating Centre for Disease Epidemiology Analytics on HIV/AIDS, Sexually Transmitted Infections, and Viral Hepatitis, Weill Cornell Medicine–Qatar, Cornell University, Qatar Foundation – Education City, Doha, Qatar; 4 Department of Population Health Sciences, Weill Cornell Medicine, Cornell University, New York, New York, USA; 5 Hamad Medical Corporation, Doha, Qatar; 6 College of Health Sciences, QU Health, Qatar University, Doha, Qatar; 7 Biomedical Research Center, Qatar University, Doha, Qatar; 8 Department of Biomedical Science, College of Health Sciences, Member of QU Health, Qatar University, Doha, Qatar; 9 Primary Health Care Corporation, Doha, Qatar; 10 Ministry of Public Health, Doha, Qatar

## Abstract

**Background:**

Mathematical modeling constitutes an important tool for planning robust responses to epidemics. This study was conducted to guide the Qatari national response to the severe acute respiratory syndrome coronavirus 2 (SARS-CoV-2) epidemic. The study investigated the epidemic’s time-course, forecasted health care needs, predicted the impact of social and physical distancing restrictions, and rationalized and justified easing of restrictions.

**Methods:**

An age-structured deterministic model was constructed to describe SARS-CoV-2 transmission dynamics and disease progression throughout the population.

**Results:**

The enforced social and physical distancing interventions flattened the epidemic curve, reducing the peaks for incidence, prevalence, acute-care hospitalization, and intensive care unit (ICU) hospitalizations by 87%, 86%, 76%, and 78%, respectively. The daily number of new infections was predicted to peak at 12 750 on May 23, and active-infection prevalence was predicted to peak at 3.2% on May 25. Daily acute-care and ICU-care hospital admissions and occupancy were forecast accurately and precisely. By October 15, 2020, the basic reproduction number *R_0_* had varied between 1.07-2.78, and 50.8% of the population were estimated to have been infected (1.43 million infections). The proportion of actual infections diagnosed was estimated at 11.6%. Applying the concept of *R_t_* tuning, gradual easing of restrictions was rationalized and justified to start on June 15, 2020, when *R_t_* declined to 0.7, to buffer the increased interpersonal contact with easing of restrictions and to minimize the risk of a second wave. No second wave has materialized as of October 15, 2020, five months after the epidemic peak.

**Conclusions:**

Use of modeling and forecasting to guide the national response proved to be a successful strategy, reducing the toll of the epidemic to a manageable level for the health care system.

Mathematical modeling has become a fundamental tool to guide surveillance of infectious diseases and emergency responses to epidemics [1-3]. Powered by surveillance and outbreak data, infection transmission models help monitor and predict epidemiological trends using real-time estimation of key indicators, such as incidence of infection, severe and critical disease cases, disease mortality, and basic reproduction number (*R_0_*; the number of secondary infections each infection would generate in a fully susceptible population) [3,4].

Qatar is a peninsula located in the Arabian Gulf, with a diverse population of 2.8 million people [5]. Like other countries, Qatar has been affected by the severe acute respiratory syndrome coronavirus 2 (SARS-CoV-2) pandemic [6-12]. Yet, the nation mounted an evidence-informed national response, in which in addition to early case identification, isolation, and quarantine through contact tracing, diverse standardized and centralized sources of data were generated, including population-based surveys. This wealth of data provided a special opportunity to understand infection transmission dynamics, predict health care needs associated with the resulting disease, coronavirus disease 2019 (COVID-19) [13], and to inform the global epidemiology of this infection.

Qatar has a unique socio-demographic structure that affected the transmission patterns of SARS-CoV-2 [8,10,12], a respiratory infection that propagates through social networks. Nearly 90% of the population are expatriates [5,14,15] with craft and manual workers (CMWs) constituting 60% of the population [16]. Of the national subpopulations, Indians (28%) constitute the largest population segment [15], followed by Bangladeshis (13%) [15], Nepalese (13%) [15], Qataris (11%) [15], Egyptians (9%) [15], and Filipinos (7%) [15]. The CMW population is predominantly male, single, and young, with the top three countries of origin being India, Bangladesh, and Nepal [16]. Most CMWs live in shared housing accommodations akin to dormitories [17].

This study was conducted to describe SARS-CoV-2 transmission dynamics in Qatar and to craft a national response using mathematical modeling of the epidemic time-course, predicting the impact of social and physical distancing restrictions and the impact of easing those restrictions, and forecasting health care needs, in terms of hospitalizations requiring acute-care and intensive care unit (ICU) beds. The study was possible thanks to a close collaborative partnership between Qatar’s Ministry of Public Health, Hamad Medical Corporation, and Weill Cornell Medicine-Qatar, and in collaboration with other national institutions such as the Primary Health Care Corporation, Qatar Biobank, and Qatar University. The study was initiated on February 20, 2020, before the identification of the first laboratory-confirmed case of community transmission on March 6, 2020, and has continued to provide real-time projections and forecasts, up to October 31, 2020 in the present study, using empirical data for SARS-CoV-2 infection for the period extending from February 5 to October 31, 2020.

The overarching aim of the present article was to provide the technical tools and a “case study” to demonstrate how individual countries can use mathematical modeling to effectively craft national public-health responses and to formulate evidence-based policy decisions that minimize the epidemic’s toll on morbidity, mortality, societies, and economies.

## METHODS

### Mathematical model

Building on our previously developed models [8,18-21], an age-structured, meta-population, deterministic mathematical model was constructed to describe SARS-CoV-2 transmission dynamics and disease progression (Figure S1 in the **Online Supplementary Document**). The model stratified the Qatari population into groups (“compartments”) according to the major nationality groups (Indians, Bangladeshis, Nepalese, Qataris, Egyptians, Filipinos, and all other nationalities), age group by decile, infection status (infected, uninfected), severity of illness (asymptomatic/mild, severe, critical), and disease/hospitalization stage (severe, critical), using sets of coupled, nonlinear, differential equations. A detailed description of the model is available in Appendix S1 of the **Online Supplementary Document**.

The risk of acquiring the infection varied between susceptible populations based on nationality, infectious contact rate per day, age-specific exposure/susceptibility to the infection, and subpopulation-mixing and age-mixing matrices parametrizing the mixing between individuals in different nationality and age groups. Following a latency period, infected individuals in the model develop an asymptomatic/mild, severe, or critical infection. The age-dependence of the proportions of infected persons developing asymptomatic/mild, severe, or critical infections was based on the modeled SARS-CoV-2 epidemic in France [22]. Severe and critical infections progress to severe and critical disease, respectively, prior to recovery. Patients are hospitalized in acute-care and ICU-care beds, respectively, based on existing standards of care in Qatar. Critical disease cases have an additional risk of COVID-19 mortality.

The model was parameterized using the best available data for SARS-CoV-2 natural history and epidemiology. A detailed description of model parameters, definitions, values, and justifications is found in Tables S1-S2 in the **Online Supplementary Document**. The size and demographic structure of the population of Qatar were based on a population census conducted by Qatar’s Planning and Statistics Authority [5]. Life expectancy was obtained from the United Nations World Population Prospects database [23].

### Model fitting and analyses

The model was fitted to the standardized and centralized databases of SARS-CoV-2 testing, infections, hospitalizations, and mortality extending from February 5 to October 31, 2020 [8], as well as to findings of ongoing epidemiologic studies [8,10,24]. Data included: 1) time-series of the number of polymerase chain reaction (PCR)-confirmed SARS-CoV-2 cases, 2) time-series of the SARS-CoV-2 testing PCR positivity rate in each national subpopulation, 3) time-series of the PCR positivity rate in symptomatic patients with suspected SARS-CoV-2 infection coming to primary health care centers, 4) time-series of the proportion of laboratory-confirmed SARS-CoV-2 cases aged >60 years, 5) time-series of new/daily hospital admissions in acute-care beds and ICU-care beds, 6) the proportion of acute-care cases subsequently transferred to ICUs, 7) time-series of hospital occupancy in acute-care and ICU-care beds, 8) the cumulative number of deaths (not time series, due to the relatively small number of deaths), 9) a community survey assessing active-infections using PCR, 10) age-distribution of antibody positivity [8,10,24], and 11) national subpopulation distribution of antibody positivity [8,10,24]. A nonlinear least-square data fitting method, based on the Nelder-Mead simplex algorithm, was used to conduct the model fitting [25].

Model fitting was used to estimate, up to October 31, 2020, epidemiologic indicators such as incidence, prevalence, attack rate (proportion of the population ever infected), and *R_0_*, as well as to forecast acute-care and ICU-care hospital admissions and hospital bed occupancy. The model was further used to evaluate the impact of implemented social and physical distancing restrictions by comparing model projections of the actual epidemic to those in a counter-factual scenario in which such interventions were not enforced. Informed by global estimates of *R_0_* in the early epidemic that ranged between 2-4 [26,27], the counter-factual scenario with no interventions was implemented assuming *R_t_* = 3. The model was also used to predict the impact of different scenarios for easing of social and physical distancing restrictions.

### Uncertainty analysis

Five hundred simulation runs were conducted to determine the range of uncertainty attending model predictions. At each run, Latin Hypercube sampling was applied in selecting input parameter values [28,29] from pre-specified ranges that assume ±30% uncertainty around parameter point estimates. The model was then refitted to input data. The resulting distribution for each model prediction, based on the 500 runs, was used to derive the mean and 95% uncertainty interval (UI).

Mathematical modeling analyses were conducted in MATLAB R2019a (Boston/MA/USA) [30] whereas statistical analyses were performed in STATA/SE 16.1 (Stata Corp, College Station, TX, USA) [31].

## RESULTS

**Figure 1:**
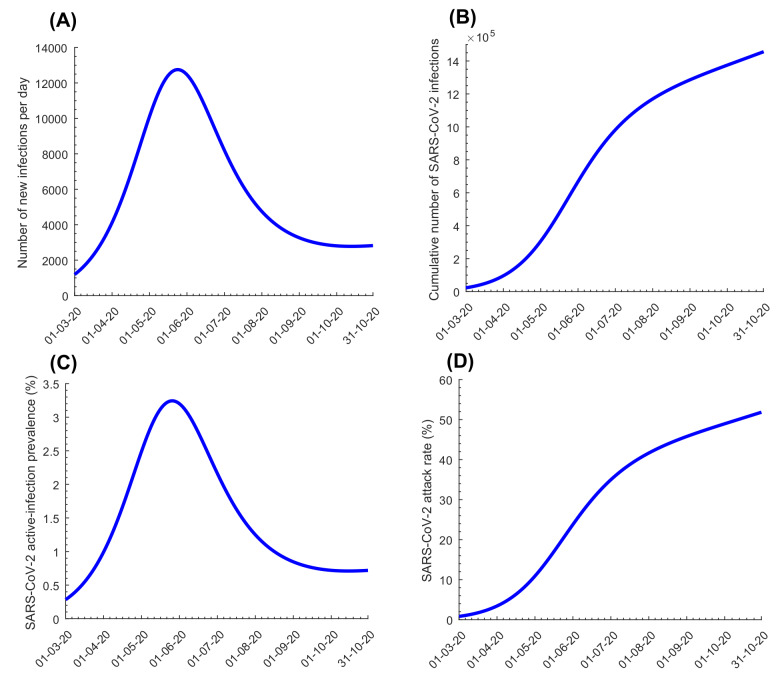
Model predictions for evolution of SARS-CoV-2 infections in the total population of Qatar. **Panel A.** Incidence (number of daily new infections). **Panel B.** Cumulative number of infections. **Panel C.** Active-infection prevalence (those latently infected or infectious). **Panel D.** Attack rate (proportion ever infected).

The model fitted the various data sources (examples in Figures S2-S3 in the **Online Supplementary Document**). **Figure 1** shows model predictions for evolution of SARS-CoV-2 incidence, cumulative incidence, active-infection prevalence, and attack rate in the total population. Peak incidence was estimated at 12 750 new infections on May 23, 2020 while peak prevalence was estimated at 3.2% on May 25, 2020. By October 15, 2020, an estimated 1 426 500 infections were projected to have occurred, for a proportion of the population infected of 50.8%. Also by October 15, 2020, the proportion of all infections that had actually been diagnosed and confirmed by PCR was estimated at 11.6%. *R_0_* varied between 1.07-2.78 from March 1 to October 15, with the highest values reached well after the onset of easing of restrictions on June 15, 2020 (Figure S4A in the **Online Supplementary Document**).

**Figure 2:**
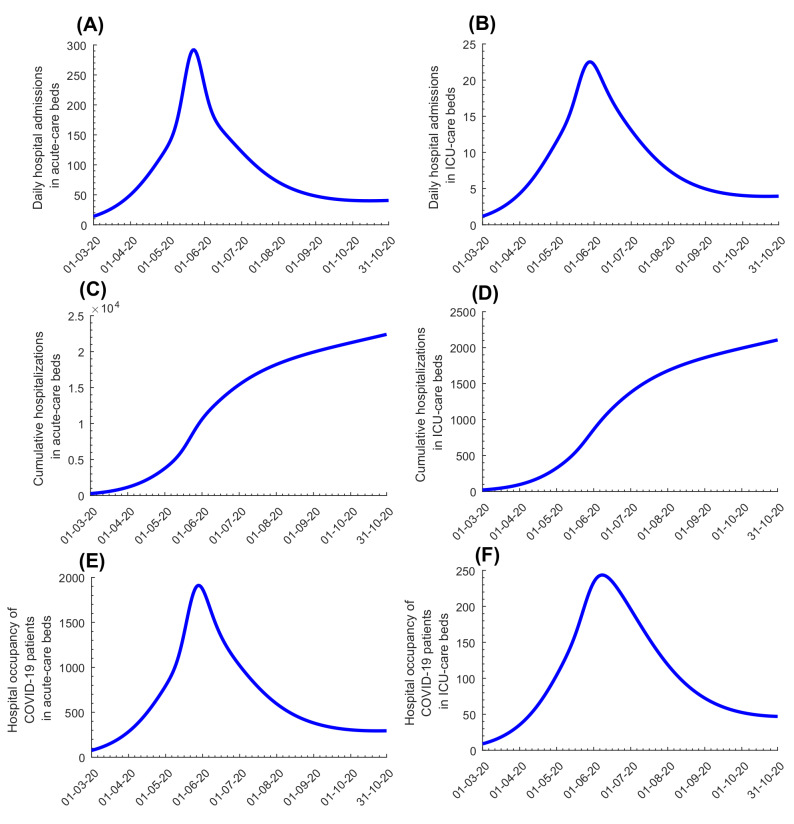
Model predictions for evolution of COVID-19 disease cases. **Panel A.** Daily hospital admissions in acute-care beds. **Panel B.** Daily hospital admissions in ICU-care beds. **Panel C.** Cumulative number of hospitalizations in acute-care beds. **Panel D.** Cumulative number of hospitalizations in ICU-care beds. **Panel E.** Hospital occupancy of COVID-19 patients (number of beds occupied at any given time) in acute-care beds. **Panel F.** Hospital occupancy of COVID-19 patients (number of beds occupied at any given time) in ICU-care beds.

**Figure 2**, Panels A-B shows model-predicted daily hospital admissions in acute-care and ICU-care beds, respectively. New hospital admissions were predicted to peak at 292 acute-care beds on May 22, 2020 and 23 ICU-care beds on May 27, 2020. **Figure 2**, Panels C-D shows evolution of hospital occupancy in acute-care and ICU-care beds. Peaks were predicted at 1910 acute-care beds on May 27, 2020 and 244 ICU-care beds on June 6, 2020. The average hospital stay in an acute-care bed was estimated at 7.7 days while the stay in an ICU-care bed was estimated at 14.0 days. These model predictions agreed with actual COVID-19 hospital admission data (Figure S3 in the **Online Supplementary Document**).

**Figure 3:**
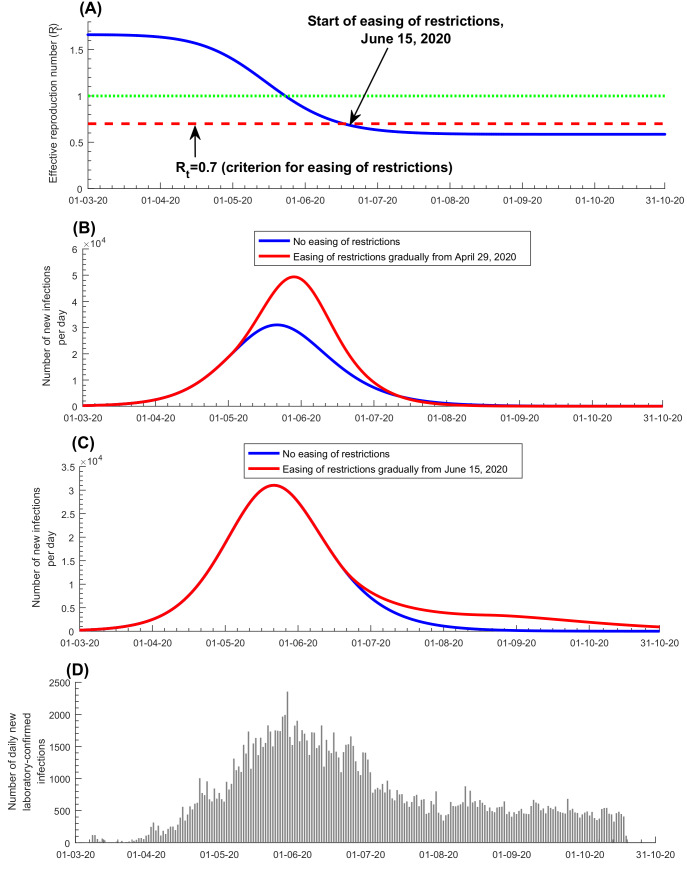
Rationale and criteria used for the start of easing of social and physical distancing restrictions. Panels A-C show the model fit and results *at the time* when the policy decision was actually made. An updated prediction for *R_t_* is in Figure S4 of the **Online Supplementary Document****. Panel A.** Effective reproduction number *R_t_* and easing of social and physical distancing restrictions. **Panel B.** Prediction of the number of daily new infections with early easing of restrictions, three weeks before the epidemic peak. **Panel C.** Prediction of the number of daily new infections with delayed easing of restrictions, three weeks after the epidemic peak. **Panel D.** The number of daily new diagnosed and laboratory-confirmed infections.

Discussions with policymakers to plan easing of social and physical distancing restrictions were initiated in April of 2020. The *effective* reproduction number (*R_t_*), the number of secondary infections each infection is generating at a given time, *t*, heavily influenced these discussions. Based on the model-predicted evolution of *R_t_ at that time* (**Figure 3**, Panel A), it was advised that no easing of restrictions should occur before the epidemic peak, then predicted to occur on May 20, as the epidemic was still in its exponential growth phase (*R_t_*>1). Model simulations confirmed that premature easing of restrictions would result in epidemic amplification (**Figure 3**, Panel B). To minimize the likelihood of a second wave and to buffer against a potential increased contact rate in the population, it was advised that easing of restrictions should not start before *R_t_* reached 0.70, and that easing of restrictions should be implemented gradually over at least two months. Model simulations confirmed this rationale, and indicated that gradual easing of restrictions after *R_t_* reached 0.70 would minimize the risk of a second wave (**Figure 3**, Panel C). Accordingly, policymakers planned and subsequently implemented a gradual easing of restrictions starting June 15, 2020, the day on which *R_t_* was predicted to decline to 0.7. This line of analysis and rationale proved successful, as no second wave had materialized as of October 15, 2020, five months after the epidemic peak (**Figure 3**, Panel D).

**Figure 4:**
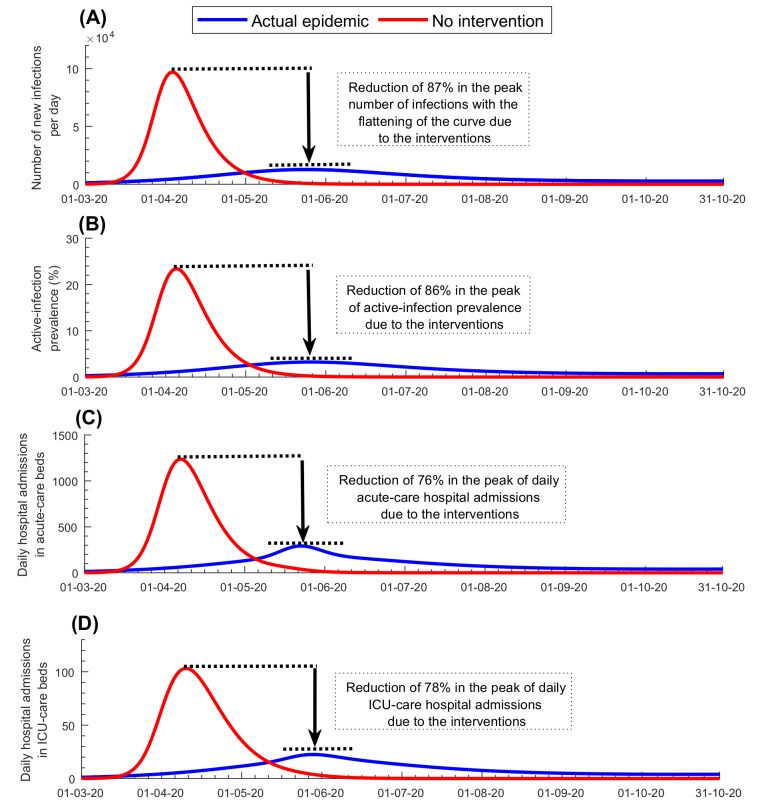
Impact of social and physical distancing interventions. **Panel A.** Number of daily new infections. **Panel B.** Active-infection prevalence (those latently infected or infectious). **Panel C.** Daily hospital admissions in acute-care beds. **Panel D.** Daily hospital admissions in ICU-care beds.

**Figure 4** and Figure S5 in the **Online Supplementary Document** show the predicted evolution of the epidemic in the *counter-factual scenario* of no social and physical distancing interventions. In the absence of these interventions, the epidemic would have peaked at 97 100 new infections per day on April 3, 2020 (**Figure 4**, Panel A), and at a prevalence of 23.4% on April 5, 2020 (**Figure 4**, Panel B). New hospital admissions would have peaked at 1235 acute-care bed admissions on April 7, 2020 (**Figure 4**, Panel C) and at 103 ICU-care bed admissions on April 10, 2020 (**Figure 4**, Panel D). Accordingly, by October 15, 2020, the enforced social and physical distancing restrictions reduced the peaks for incidence, prevalence, and acute-care and ICU-care hospital admissions by >75% (**Figure 4**, Panels A-D), and averted 840 000 infections (37%; Figure S5A in the **Online Supplementary Document**), 209 deaths (46%; Figure S5B in the **Online Supplementary Document**), 10 110 acute-care hospital admissions (32%; Figure S5C in the **Online Supplementary Document**), and 1056 ICU-care hospital admissions (34%; Figure S5D in the **Online Supplementary Document**). These results show the extent of flattening of the epidemic curve that was accomplished with the enforced social and physical distancing interventions.

Figure S6 in the **Online Supplementary Document** shows the results of the uncertainty analysis for the key epidemiological indicators in **Figure 1**, and Figure S7 in the **Online Supplementary Document** shows the results of the uncertainty analysis for the key hospitalization indicators in **Figure 2**. The results indicated overall narrow uncertainty intervals confirming the model’s predictive power.

## DISCUSSION

Our study demonstrates that mathematical modeling was influential in informing the national public-health response and in formulating evidence-based policy decisions to minimize the pandemic’s toll on health, society, and the economy. The model, which was implemented in real-time, starting from late February 2020, and was continuously updated and refined as more data became available, predicted with reasonable accuracy and precision the key epidemiologic indicators, such as the epidemic peak and the impact of easing of restrictions, as well as health care needs, at a time of uncertainty in which knowledge of the epidemiology of this infection was growing but still limited.

One of the highlights of this modeling approach is the application of the concept of *rational R_t_* *tuning* for managing the easing of restrictions (**Figure 4**). Grounded on a theoretical foundation [4], rational *R_t_* tuning proved to be a successful and effective strategy in safely easing the restrictions so as to ensure social and economic stability and functionality, while minimizing the risk of a second wave (**Figure 3**). Another highlight is the estimation of health care needs that guided resource-allocation planning well before the time when these resources were needed. Throughout the epidemic, including the epidemic peak, health care needs in Qatar remained well within the health system capacity, avoiding any serious strain. Importantly, this forecasting of health care needs also prevented resource waste by avoiding overestimation of health care needs.

Despite the large number of infections in Qatar, results show that the epidemic would have been far worse if no social and physical distancing interventions had been enforced. In absence of interventions, the epidemic would have progressed very rapidly to a peak nearly 10-fold higher than what was actually observed (**Figure 4**). Disease burden would have been much larger and the health care system would have been strained to the point of collapse. This demonstrates that for a respiratory infection with such large *R_t_* and serious disease sequalae, inaction would have had dire consequences, and that the national strategy focused on flattening the epidemic curve was appropriate to manage the epidemic.

An important finding of this study is that PCR-confirmed infections constitute only a small fraction of the actual number of infections. Only 11.6% of infections were estimated to have ever been diagnosed, probably because most infections were asymptomatic or mild. Indeed, a nation-wide population-based survey in Qatar showed that 58.5% of those who were PCR positive in this survey reported no symptoms during the last two weeks preceding the survey [8]. The growing number of serological testing studies in Qatar have also shown that the vast majority of those who are antibody-positive were never diagnosed with this infection [8,10,12,24]. For instance, out of all those antibody-positive in a nation-wide seroprevalence survey of the CMW population, only 9.3% had a documented, PCR-confirmed infection *prior* to antibody testing [10], affirming that as estimated by the model, nine of every 10 infections were never diagnosed. These findings are also consistent with a growing body of serological evidence from other countries [32-36]. Of note that the latter further suggests high variability in exposure to the infection across countries [32-36].

We found that >97% of infections estimated to have occurred did not require hospitalization. The low infection severity appears to be a consequence of the young age profile of the population, with only 2% being >60 years of age [5,8,11,19], in addition to a well-funded health care system that emphasizes a proactive, high-quality standard of care [8], and possibly high levels of T cell cross-reactivity against SARS-CoV-2, reflecting T cell memory of circulating ‘common cold’ coronaviruses [37-41].

This study has limitations. Model estimates are contingent on the validity and generalizability of input data. Our estimates were based on current SARS-CoV-2 natural history and disease progression parameters, but our understanding of this infection is still evolving. We modeled the age-specific distributions for infection severity, criticality, and mortality using relative risk data from the SARS-CoV-2 epidemic in France [22], which may not extend to Qatar. However, these estimates broadly agreed with those of a recently-completed study that estimated these relative risks specifically for Qatar [11]. Available input data were most complete at the national level. We did not have regional data or sufficient data about social networks of different national subpopulations and patterns of mixing between those subpopulations to factor them into the model. Despite these limitations, our model, tailored to the complexity of the epidemic in Qatar, was able to reproduce observed epidemic trends, and to provide useful and consequential predictions and insights about infection transmission and health care needs. Importantly, the modeling estimates successfully influenced the national response.

In conclusion, Qatar experienced a large SARS-CoV-2 epidemic, but avoided a burdensome epidemic, such as that unfolding in other counties. Mathematical modeling played an influential role in guiding the national public-health response by characterizing and understanding the epidemic, forecasting health care needs, predicting the impact of social and physical distancing restrictions, and rationalizing and justifying the easing of restrictions. While this article illustrates a successful case study, the modeling tools employed here can be adapted and applied in other countries to guide SARS-CoV-2 epidemic control, preparedness for the current or future waves of infection, or enforcement and easing of restrictions or other interventions, such as vaccination [21].

## Additional material

Online Supplementary Document
