# Supplementary material for: Mathematical modeling of the SARS-CoV-2 epidemic in Qatar and its impact on the national response to COVID-19
Source: J Glob Health. 2021 Jan 16;11:05005. doi: 10.7189/jogh.11.05005 (PMC7897910; doi:10.7189/jogh.11.05005)
Supplement: Online Supplementary Document [file jogh-11-05005-s001.pdf]

Supplementary Material

Mathematical modeling of the SARS-CoV-2 epidemic in Qatar and its impact on the national response to COVID-19

I. SARS-CoV-2 mathematical model structure

A deterministic age-structured meta-population compartmental model was developed to describe the severe acute respiratory syndrome coronavirus 2 (SARS-CoV-2) transmission dynamics and disease progression in the population of Qatar, factoring subpopulation heterogeneity in exposure to the infection. The model stratified the population into compartments according to nationality subpopulation, age group (0-9, 10-19, 20-29,…, ≥80 years), infection status (infected, uninfected), infection stage (asymptomatic/mild, severe, critical), and disease stage (severe disease or critical disease). All Coronavirus Disease 2019 (COVID-19) mortality was assumed to occur in individuals that are in the critical disease stage. The model is based on extension and adaptation of our calibrated mathematical models developed to characterize SARS-CoV-2 transmission dynamics [1-5].

Epidemic dynamics were described using a system of coupled nonlinear differential equations for each age group and subpopulation (nationality) group. Each age group, a, denoted a ten-year age band apart from the last category which grouped together all individuals ≥80 years of age. The population was divided into seven resident subpopulation groups i $\left(i=1,2,3,4,5,6,7\right)$ representing the subpopulations of Indians, Bangladeshis, Nepalese, Qataris, Egyptians, Filipinos, and all other nationalities, respectively—these are the largest nationality subpopulation groups in Qatar. Qatar's population composition and subpopulations size and demographic structure were based on findings of "The Simplified Census of Population, Housing, and Establishments" conducted by Qatar's Planning and Statistics Authority [6]. Life expectancy was obtained from the United Nations World Population Prospects database [7].

Figure S1. Schematic diagram describing the basic structure of the SARS-CoV-2 mathematical model.

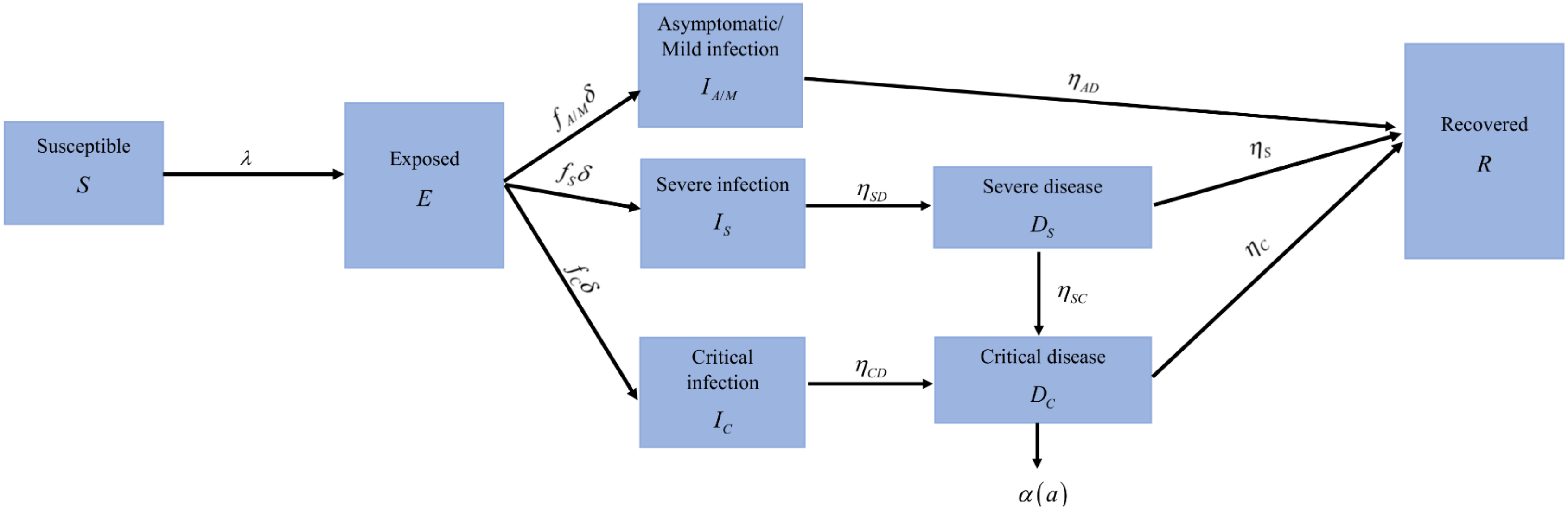

The model was expressed in terms of the following system of coupled nonlinear differential equations for each subpopulation group and age group:

$$\frac{dS(a,i)}{dt} = \xi(a-1)S(a-1,i) - \left(\lambda(a,i) + \mu + \xi(a)\right)S(a,i)$$
$$\frac{dE(a,i)}{dt} = \xi(a-1)E(a-1,i) + \lambda(a,i)S(a,i) - \left(\delta + \mu + \xi(a)\right)E(a,i)$$
$$\frac{dI_{A/M}(a,i)}{dt} = \xi(a-1)I_{A/M}(a-1,i) + f_{A/M}(a)\delta E(a,i) - \left(\eta_{AD} + \mu + \xi(a)\right)I_{A/M}(a,i)$$
$$\frac{dI_S(a,i)}{dt} = \xi(a-1)I_S(a-1,i) + f_S(a)\delta E(a,i) - \left(\eta_{SD} + \mu + \xi(a)\right)I_S(a,i)$$
$$\frac{dI_C(a,i)}{dt} = \xi(a-1)I_C(a-1,i) + f_C(a)\delta E(a,i) - \left(\eta_{CD} + \mu + \xi(a)\right)I_C(a,i)$$
$$\frac{dD_S(a,i)}{dt} = \xi(a-1)D_S(a-1,i) + \eta_{SD}I_S(a,i) - \left(\eta_S + \eta_{SC} + \mu + \xi(a)\right)D_S(a,i)$$
$$\frac{dD_C(a,i)}{dt} = \xi(a-1)D_C(a-1,i) + \eta_{CD}I_C(a,i) + \eta_{SC}D_S(a,i) - \left(\eta_C + \mu + \xi(a) + \alpha(a)\right)D_C(a,i)$$
$$\frac{dR(a,i)}{dt} = \xi(a-1)R(a-1,i) + \eta_{A/M}I_{A/M}(a,i) + \eta_S D_S(a,i) + \eta_C D_C(a,i) - \left(\mu + \xi(a)\right)R(a,i)$$

The definitions of population variables and symbols used in the equations are in Table S1.

Table S1. Definitions of population variables and symbols used in the model

Symbol	Definition
$S(a,i)$	Susceptible population
$E(a,i)$	Latently infected population
$I_{A/M}(a,i)$	Population with asymptomatic/mild infection
$I_S(a,i)$	Population with severe infection
$I_C(a,i)$	Population with critical infection
$D_S(a,i)$	Population with hospitalization in acute-care beds
$D_C(a,i)$	Population with hospitalization in intensive care unit beds
$R(a,i)$	Recovered population
n_{age}	Number of age groups
n_{pop}	Number of subpopulation groups
$\xi(a)$	Transition rate from one age group to the next age group. Here $\xi(0) = \xi(n_{age}) = 0$
$\sigma(a)$	Susceptibility profile to the infection in each age group
$\psi(i)$	Level of exposure profile in each subpopulation group i
$1/\delta$	Duration of latent infection

β	Average rate of infectious contacts
$1/\eta_{AD}$	Duration of asymptomatic/mild infection infectiousness
$1/\eta_{SD}$	Duration of severe infection infectiousness before isolation and/or hospitalization
$1/\eta_{S}$	Duration of severe disease following onset of severe disease
$1/\eta_{CD}$	Duration of critical infection infectiousness before isolation and/or hospitalization
$1/\eta_{C}$	Duration of critical disease following onset of critical disease
$1/\eta_{SC}$	Rate of disease progression from severe disease to critical disease
$\alpha(a)$	Disease mortality rate in each age group
$1/\mu$	Natural death rate
$f_{A/M}(a)$	Proportion of infections that will progress to be asymptomatic/mild infections
$f_S(a)$	Proportion of infections that will progress to be infections that require hospitalization in acute-care beds
$f_C(a)$	Proportion of infections that will progress to be infections that require hospitalization in intensive care unit beds
Z	The subpopulation mixing matrix
H	The age-mixing matrix

The force of infection $\lambda(a,i)$ (hazard rate of infection) experienced by each susceptible $S(a,i)$ population, is given by

$$\lambda(a,i)=\beta\psi(i)\sigma(a)\sum_{a'=1}^{n_{age}}\sum_{i'=1}^{n_{pop}}Z_{i,i'}H_{a,a',i'}\frac{I_{A/M}(a',i')+I_S(a',i')+I_C(a',i')}{\begin{bmatrix}S(a',i')+E(a',i')+I_{A/M}(a',i')+I_S(a',i')+I_C(a',i')\\+D_S(a',i')+D_C(a',i')+R(a',i')\end{bmatrix}}$$

Here β is the rate of infectious contacts, $\psi(i)$ is the level of exposure profile in each subpopulation group i, and $\sigma(a)$ is the susceptibility profile to the infection in each age group a.

To account for temporal variation in the basic reproduction number (R_0), we incorporated temporal changes in the rate of infectious contacts. We parameterized the temporal variation (time dependence of β) through the following combined function of the Woods-Saxon and logistic functions.

$$\beta(t) = \underbrace{\frac{a_1}{1+e^{\left(\frac{t-b_1}{c_1}\right)}}}_{\textit{Wood – Saxon function}} + \underbrace{\frac{a_2}{1+e^{-\left(\frac{t-b_2}{c_2}\right)}}}_{\textit{Logistic function}}$$

This function was mathematically designed to describe and characterize the time evolution of the level of risk of exposure before and after easing of restrictions. It was informed by our knowledge of SARS-CoV-2 epidemiology in Qatar [4], and it provided a robust fit to the data. Here a_1, a_2, b_1, b_2, c_1, and c_2 are fitting parameters.

The mixing among the different age groups and subpopulation groups is dictated by the mixing matrices $H_{a,a',i'}$ (for age group mixing) and $Z_{i,i'}$ (for subpopulation group mixing). These matrices provide the likelihood of mixing and are given by the following expressions:

$$H_{a,a',i'} = e_{Age}\delta_{a,a'} + (1-e_{Age})\frac{\begin{array}{c} S(a',i')+E(a',i')+I_{A/M}(a',i')+I_S(a',i')+I_C(a',i') \\ +D_{A/M}(a',i')+D_S(a',i')+D_C(a',i')+R(a',i') \end{array}}{\sum_{a''=1}^{n_{age}}\left[\begin{array}{l} S(a'',i')+E(a'',i')+I_{A/M}(a'',i')+I_S(a'',i')+I_C(a'',i') \\ +D_{A/M}(a'',i')+D_S(a'',i')+D_C(a'',i')+R(a'',i') \end{array}\right]}$$

$$Z_{i,i'} = \delta_{i,i'} + n_{pop} q W, \quad \text{where } \mathrm{W}=\begin{cases} 0 & \text{if } i = i' \\ \dfrac{1}{n_{pop}\left(n_{pop}-1\right)} & \text{if } i \neq i' \end{cases}$$

Here, $\delta_{a,a'}$ (and $\delta_{i,i'}$) is the identity matrix. $e_{Age} \in \left[0,1\right]$ measures the degree of assortativeness in the age mixing. At the extreme $e_{Age} = 0$, the mixing is fully proportional, while at the other extreme, $e_{Age} = 1$, the mixing is fully assortative, that is individuals mix only with members in their own age group. W is the subpopulation connectivity matrix of dimension $n_{pop} \times n_{pop}$ and the mixing is assumed symmetric for all subpopulations. The W matrix normalizes to 1, that is the

sum of all entries adds up to 1. q parametrizes the connectivity to other subpopulations relative to the connectivity within the same subpopulation.

II. Model fitting and parameter values

The model was fitted to the following sources of data: 1) time-series of the number of polymerase chain reaction (PCR) laboratory-confirmed SARS-CoV-2 cases, 2) time-series of SARS-CoV-2 testing PCR positivity rate in each nationality subpopulation, 3) time-series of PCR positivity rate in symptomatic patients with suspected SARS-CoV-2 infection presenting to primary healthcare centers, 4) time-series of proportion of laboratory-confirmed SARS-CoV-2 cases aged >60 years, 5) time-series of new/daily hospital admissions in acute-care beds and in ICU-care beds, 6) proportion of acute-care bed cases transferred subsequently to ICU-care beds, 7) time-series of hospital occupancy in acute-care beds and in ICU-care beds, 8) cumulative number of deaths (not time series with the relatively small number of deaths), 9) one community survey assessing active-infection using PCR, 10) age-distribution of antibody positivity [4,8,9], and 11) nationality subpopulation distribution of antibody positivity [4,8,9].

Model input parameters were based on best available empirical data for SARS-CoV-2 natural history and epidemiology. Model parameter values are listed in Table 2. The following parameters were derived by fitting the model to data: f_C, η_S, η_C, η_{SC}, α, $\sigma(a)$, $\psi(i)$, e_{Age}, q, a_0, a_1, a_2, b_0, b_1, b_2, t_0, c_1, and c_2.

Table S2. Model parameter values.

Parameter	Symbol	Value	Justification
Duration of latent infection	$1/\delta$	3.69 days	Based on existing estimate [10] and based on a median incubation period of 5.1 days [11] adjusted by observed viral load among infected persons [12] and reported transmission before onset of symptoms [13].

Duration of infectiousness	$1/\eta_{AD}$<br>$1/\eta_{SD}$<br>$1/\eta_{CD}$	3.48 days	Based on existing estimate[10] and based on observed time to recovery among persons with mild infection [10,14] and observed viral load in infected persons [12,13,15].
Life expectancy in Qatar	$1/\mu$	80.7 years	United Nations World Population Prospects database [7].
Disease mortality rate in each age group	$\alpha(a)$		The distribution and age dependence of COVID-19 mortality was based on the modeled SARS-CoV-2 epidemic in France [16].
Age 0-19 years	$RRD1\times\alpha$	$RRD1=0.1$	Model-estimated relative risk of death based on the SARS-CoV-2 epidemic in France [16].
Age 20-29 years	$RRD2\times\alpha$	$RRD2=0.4$	Model-estimated relative risk of death based on the SARS-CoV-2 epidemic in France [16].
Age 30-39 years	α	Reference category	Model fitting
Age 40-49 years	$RRD3\times\alpha$	$RRD3=3.0$	Model-estimated relative risk of death based on the SARS-CoV-2 epidemic in France [16].
Age 50-59 years	$RRD4\times\alpha$	$RRD4=10.0$	Model-estimated relative risk of death based on the SARS-CoV-2 epidemic in France [16].
Age 60-69 years	$RRD5\times\alpha$	$RRD5=45.0$	Model-estimated relative risk of death based on the SARS-CoV-2 epidemic in France [16].
Age 70-79 years	$RRD6\times\alpha$	$RRD6=120.0$	Model-estimated relative risk of death based on the SARS-CoV-2 epidemic in France [16].
Age 80+ years	$RRD7\times\alpha$	$RRD7=505.0$	Model-estimated relative risk of death based on the SARS-CoV-2 epidemic in France [16].
Proportion of infections that will progress to be infections that require hospitalization in acute-care beds	$f_S(a)$		The distribution and age dependence of asymptomatic/mild, severe, or critical infections was based on the modeled SARS-CoV-2 epidemic in France [16].
Age 0-19 years	$RRS1\times f_S(t)$	$RRS1=0.1$	Model-estimated relative risk of severe infection based on the SARS-CoV-2 epidemic in France [16].
Age 20-29 years	$RRS2\times f_S(t)$	$RRS2=0.5$	Model-estimated relative risk of severe infection based on the SARS-CoV-2 epidemic in France [16].
Age 30-39 years	$f_S(t)=a_0e^{-\left(\frac{t-t_0}{b_0}\right)^2}$	Reference category	This parameter was described by a Gaussian function, as it provided the best fit for the data of the daily hospital admissions in acute-care beds.
Age 40-49 years	$RRS3\times f_S(t)$	$RRS3=1.2$	Model-estimated relative risk of severe infection based on the SARS-CoV-2 epidemic in France [16].

Age 50-59 years	$RRS4 \times f_S(t)$	$RRS4 = 2.3$	Model-estimated relative risk of severe infection based on the SARS-CoV-2 epidemic in France [16].
Age 60-69 years	$RRS5 \times f_S(t)$	$RRS5 = 4.5$	Model-estimated relative risk of severe infection based on the SARS-CoV-2 epidemic in France [16].
Age 70-79 years	$RRS6 \times f_S(t)$	$RRS6 = 7.8$	Model-estimated relative risk of severe infection based on the SARS-CoV-2 epidemic in France [16].
Age 80+ years	$RRS7 \times f_S(t)$	$RRS7 = 27.6$	Model-estimated relative risk of severe infection based on the SARS-CoV-2 epidemic in France [16].
Proportion of infections that will progress to be infections that require hospitalization in intensive care unit beds	$f_C(a)$		The distribution and age dependence of asymptomatic/mild, severe, or critical infections was based on the modeled SARS-CoV-2 epidemic in France [16].
Age 0-19 years	$RRC1 \times f_C$	$RRC1 = 0.2$	Model-estimated relative risk of critical infection based on the SARS-CoV-2 epidemic in France [16].
Age 20-29 years	$RRC2 \times f_C$	$RRC2 = 0.3$	Model-estimated relative risk of critical infection based on the SARS-CoV-2 epidemic in France [16].
Age 30-39 years	f_C	Reference category	Model fitting
Age 40-49 years	$RRC3 \times f_C$	$RRC3 = 1.8$	Model-estimated relative risk of critical infection based on the SARS-CoV-2 epidemic in France [16].
Age 50-59 years	$RRC4 \times f_C$	$RRC4 = 4.7$	Model-estimated relative risk of critical infection based on the SARS-CoV-2 epidemic in France [16].
Age 60-69 years	$RRC5 \times f_C$	$RRC5 = 10.6$	Model-estimated relative risk of critical infection based on the SARS-CoV-2 epidemic in France [16].
Age 70-79 years	$RRC6 \times f_C$	$RRC6 = 13.6$	Model-estimated relative risk of critical infection based on the SARS-CoV-2 epidemic in France [16].
Age 80+ years	$RRC7 \times f_C$	$RRC7 = 8.7$	Model-estimated relative risk of critical infection based on the SARS-CoV-2 epidemic in France [16].

III. Basic reproduction number R_0 and effective reproduction number R_t

As informed by the method of Heffernan and *et al.* [17], the overall basic reproduction number (R_0) and overall effective reproduction number (R_t) were derived to be

$$R_0=\sum_{i=1}^{n_{pop}}\sum_{a=1}^{n_{age}}\sum_{i'=1}^{n_{pop}}\sum_{a'=1}^{n_{age}}\left(\frac{N(a,i)}{N_{Tot}}\right)\beta\psi(i)\sigma(a)Z_{i,i'}H_{a,a',i'}\left(\frac{\delta}{\delta+\mu+\xi(a')}\right)\left(\frac{1}{\eta+\mu+\xi(a')}\right)$$

$$R_t=\sum_{i=1}^{n_{pop}}\sum_{a=1}^{n_{age}}\left(\frac{S(a,i)}{\sum_{\gamma=A/M,S,C}\sum_{j=1}^{n_{pop}}\sum_{b=1}^{n_{age}}I_{\gamma}(b,j)}\right)\beta\psi(i)\sigma(a)\sum_{\tau=A/M,S,C}\sum_{i'=1}^{n_{pop}}\sum_{a'=1}^{n_{age}}Z_{i,i'}H_{a,a',i'}\left(\frac{I_{\tau}(a',i')}{N(a',i')}\right)\left(\frac{\delta}{\delta+\mu+\xi(a')}\right)\left(\frac{1}{\eta+\mu+\xi(a')}\right)$$

Here, N_{Tot} is the total population size and $N(a,i)$ is the population size of each age group a and subpopulation group i.

Figure S2. Model fits to (A) SARS-CoV-2 laboratory-confirmed cases and (B) testing PCR positivity rate.

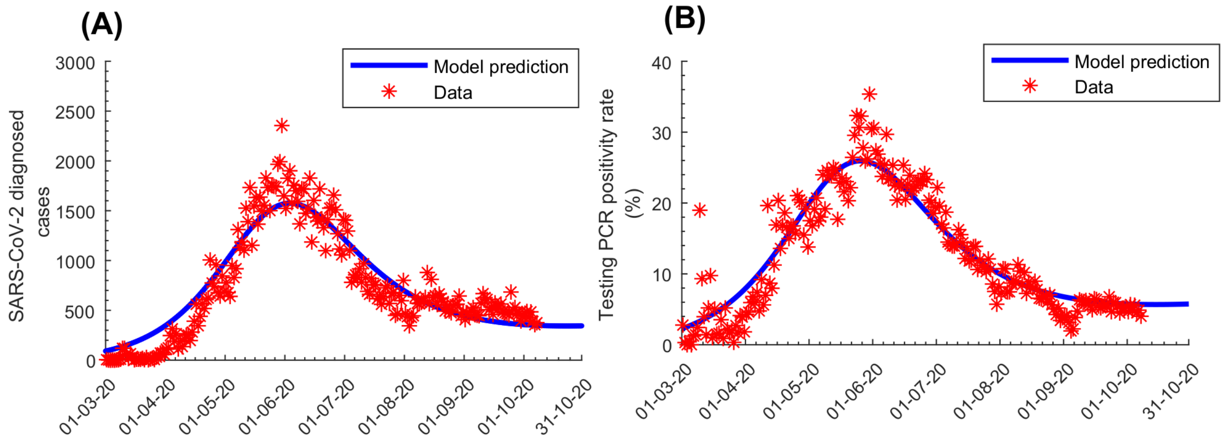

Figure S3. Model fits to A) daily hospital admissions in acute-care beds, B) daily hospital admissions in ICU-care beds, C) hospital occupancy of COVID-19 patients (number of beds occupied at any given time) in acute-care beds, and D) hospital occupancy of COVID-19 patients in ICU-care beds.

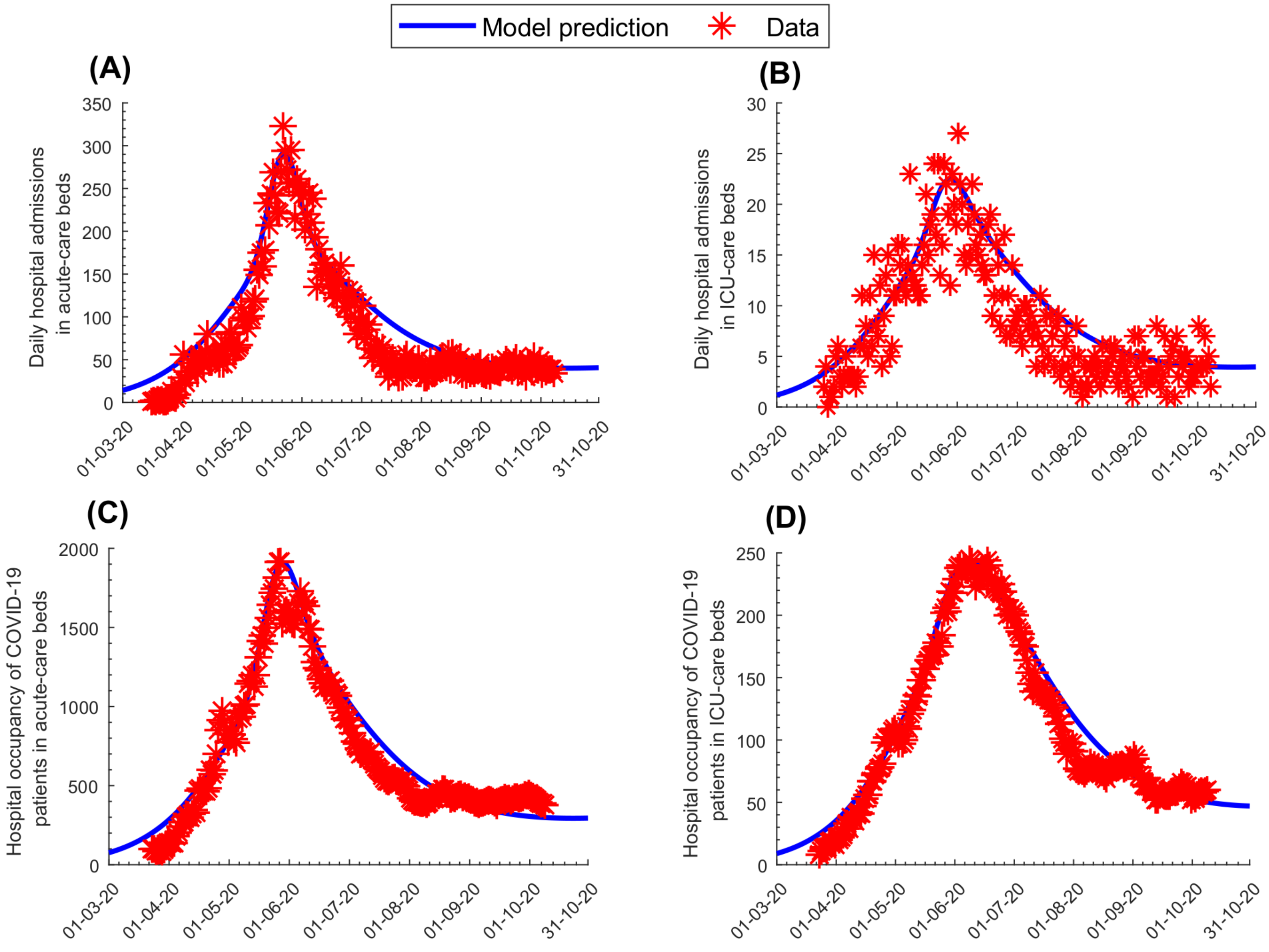

Figure S4. Evolution of the basic reproduction number R_0 (A) and effective reproduction number R_t (B) in Qatar.

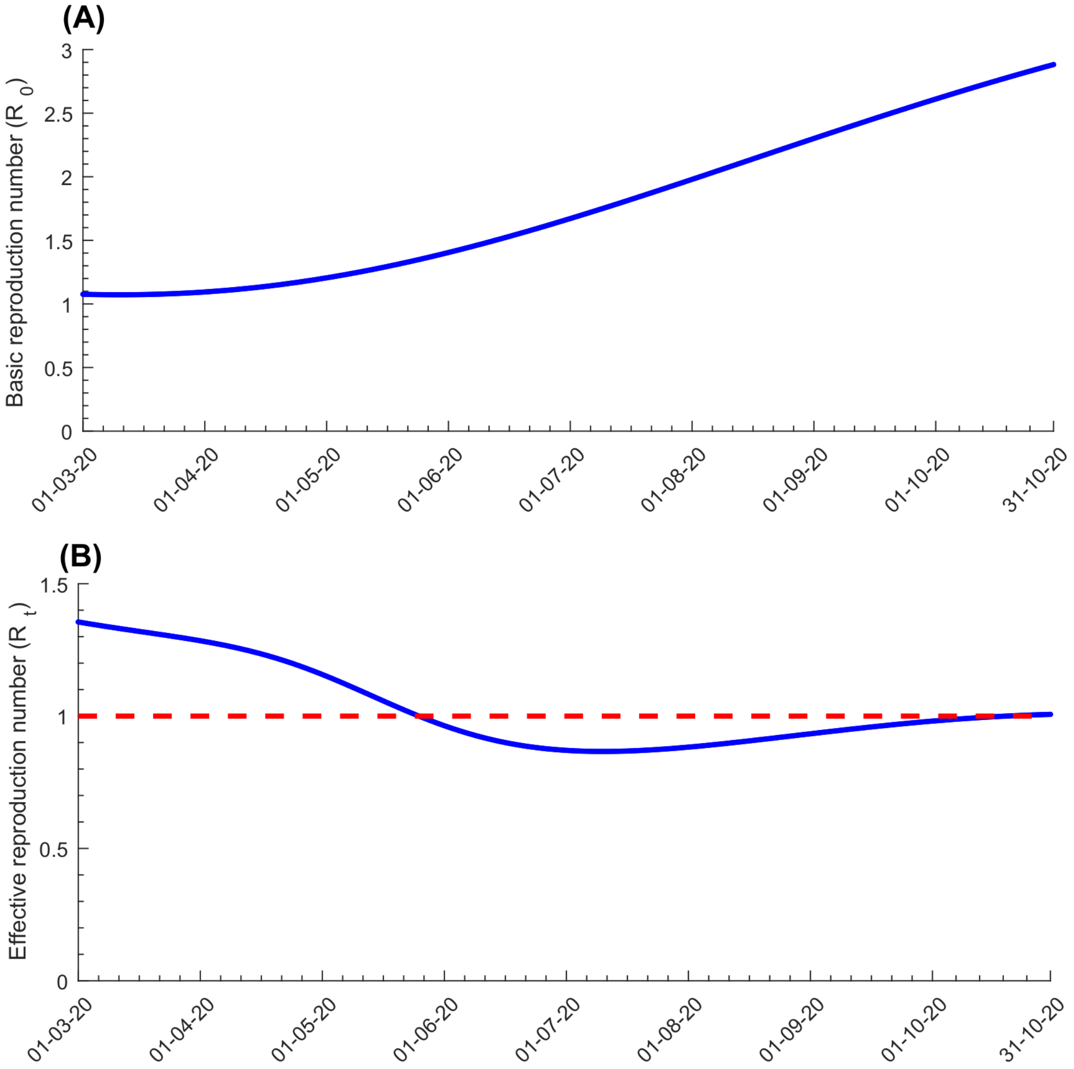

Figure S5. Impact of the social and physical distancing interventions on A) cumulative number of infections, B) cumulative number of deaths, C) cumulative number of hospital admissions in acute-care beds, and D) cumulative number of hospital admissions in ICU-care beds.

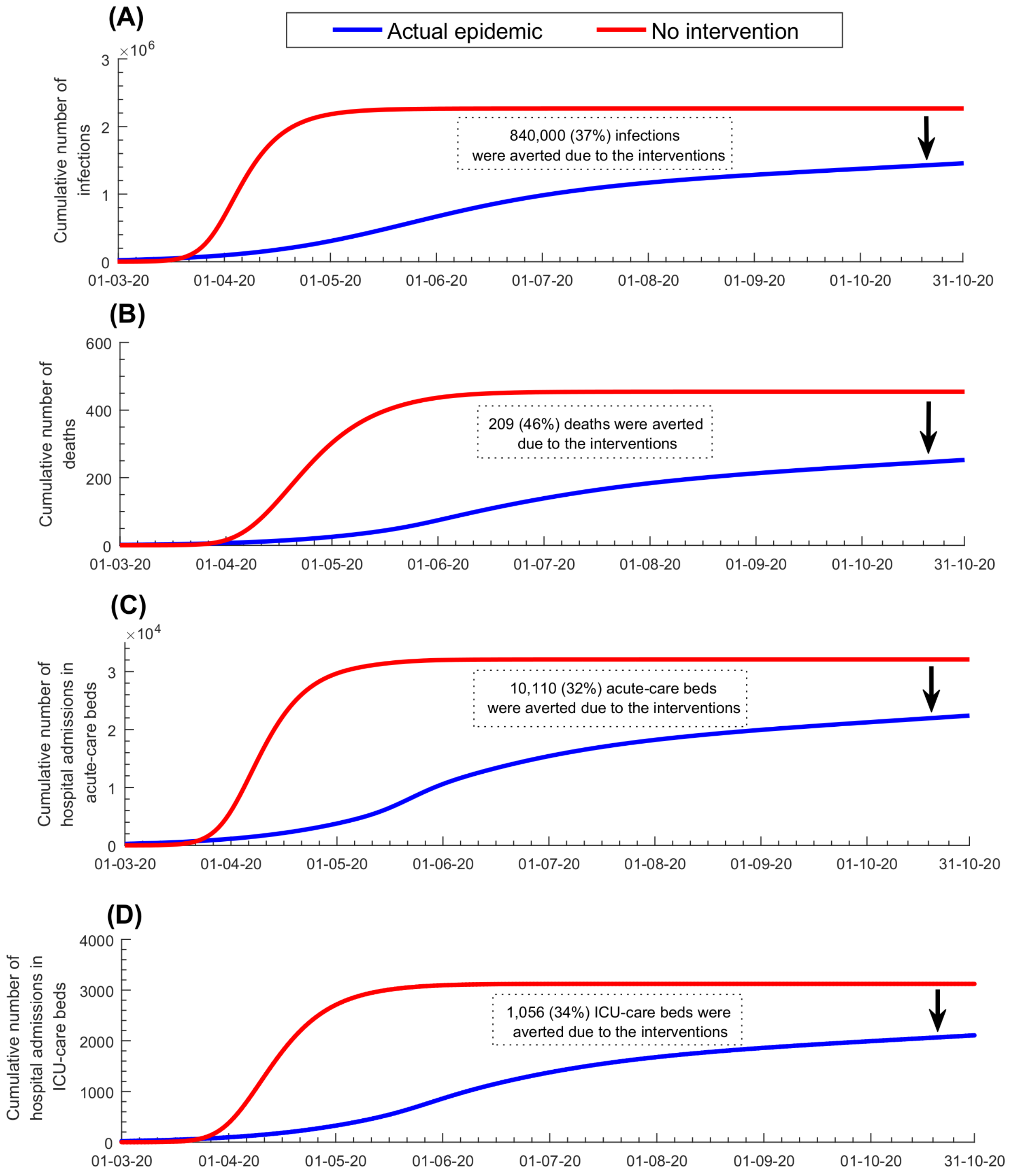

Figure S6. Uncertainty analysis. Mean and 95% uncertainty interval (UI) for the evolution of SARS-CoV-2 A) incidence (number of daily new infections), B) cumulative number of infections, C) active-infection prevalence (those latently infected or infectious), and D) attack rate (proportion ever infected), in the total population of Qatar.

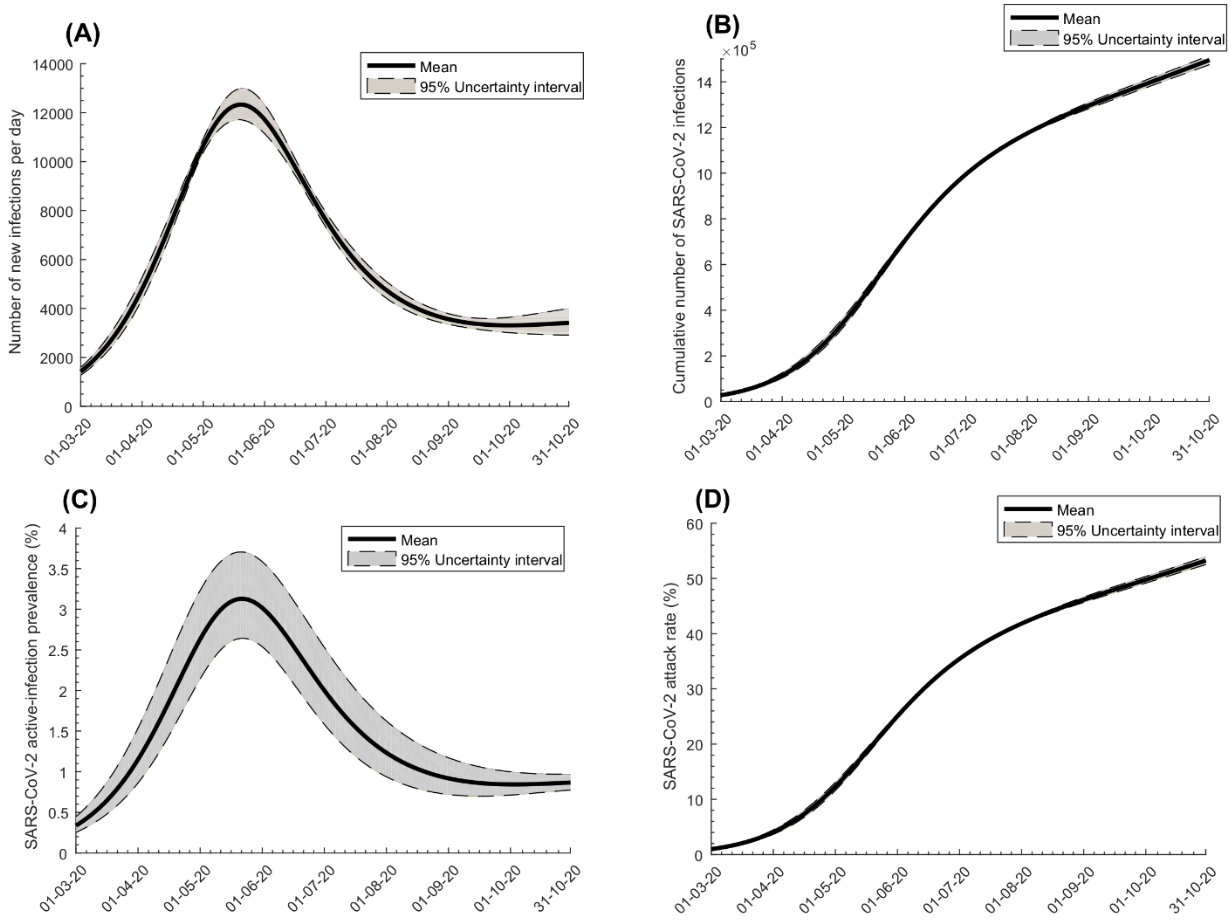

Figure S7. Uncertainty analysis. Mean and 95% uncertainty interval (UI) for the evolution of COVID-19 A) daily hospital admissions in acute-care beds, B) daily hospital admissions in ICU-care beds, C) cumulative number of hospitalizations in acute-care beds, D) cumulative number of hospitalizations in ICU-care beds, E) hospital occupancy of COVID-19 patients (number of beds occupied at any given time) in acute-care beds, and F) hospital occupancy of COVID-19 patients in ICU-care beds.

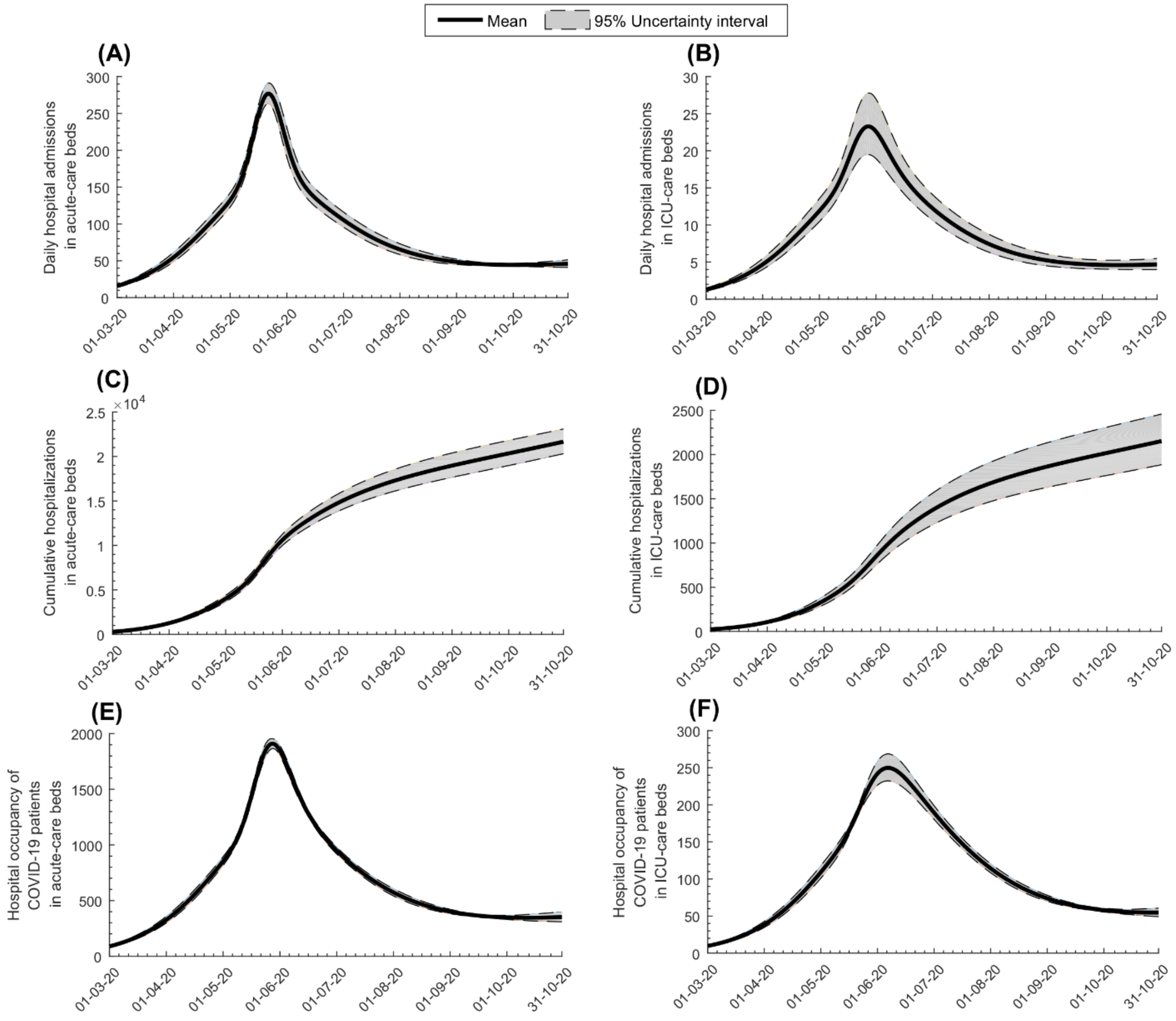

References

1 Ayoub HH, Chemaitelly H, Mumtaz GR, Seedat S, Awad SF, Makhoul M, et al. Characterizing key attributes of the epidemiology of COVID-19 in China: Model-based estimations. Global Epidemiology. 2020;100042.

2 Ayoub HH, Chemaitelly H, Seedat S, Mumtaz GR, Makhoul M, Abu-Raddad LJ. Age could be driving variable SARS-CoV-2 epidemic trajectories worldwide. Plos One. 2020;15.

3 Makhoul M., Ayoub H.H., Chemaitelly H., Seedat S., Mumtaz G.R., Al-Omari S., et al. Epidemiological impact of SARS-CoV-2 vaccination: Mathematical modeling analyses. Vaccines. 2020;8.

4 Abu-Raddad LJ, Chemaitelly H, Ayoub HH, Al Kanaani Z, Al Khal A, Al Kuwari E, et al. Characterizing the Qatar advanced-phase SARS-CoV-2 epidemic. medRxiv. 2020:2020.07.16.20155317v2 (non-peer-reviewed preprint).

5 Makhoul M, Abou-Hijleh F, Seedat S, Mumtaz GR, Chemaitelly H, Ayoub H, et al. Analyzing inherent biases in SARS-CoV-2 PCR and serological epidemiologic metrics. medRxiv. 2020:2020.08.30.20184705.

6 Planning and Statistics Authority-State of Qatar. The Simplified Census of Population, Housing & Establishments. Available from: https://www.psa.gov.qa/en/statistics/Statistical%20Releases/Population/Population/2018/Population_social_1_2018_AE.pdf Accessed on: April 2, 2020. 2019.

7 United Nations Department of Economic and Social Affairs Population Dynamics. The 2019 Revision of World Population Prospects. Available from https://population.un.org/wpp/. Accessed on March 1st, 2020. 2020.

8 Coyle P., et al. Seroprevalence of SARS-CoV-2 infection in the urban population of Qatar. under preparation.

9 Al-Thani MH, Farag E, Bertollini R, Al Romaihi HE, Abdeen S, Abdelkarim A, et al. Seroprevalence of SARS-CoV-2 infection in the craft and manual worker population of Qatar. medRxiv. 2020:2020.11.24.20237719 (non-peer-reviewed preprint).

10 Li R, Pei S, Chen B, Song Y, Zhang T, Yang W, et al. Substantial undocumented infection facilitates the rapid dissemination of novel coronavirus (SARS-CoV2). Science. 2020;368:489-93.

11 Lauer SA, Grantz KH, Bi Q, Jones FK, Zheng Q, Meredith HR, et al. The Incubation Period of Coronavirus Disease 2019 (COVID-19) From Publicly Reported Confirmed Cases: Estimation and Application. Ann Intern Med. 2020;172:577-82.

12 Zou L, Ruan F, Huang M, Liang L, Huang H, Hong Z, et al. SARS-CoV-2 Viral Load in Upper Respiratory Specimens of Infected Patients. N Engl J Med. 2020;382:1177-9.

13 Rothe C, Schunk M, Sothmann P, Bretzel G, Froeschl G, Wallrauch C, et al. Transmission of 2019-nCoV Infection from an Asymptomatic Contact in Germany. N Engl J Med. 2020;382:970-1.

14 World Health Organization. Report of the WHO-China Joint Mission on Coronavirus Disease 2019 (COVID-19). Available from :https://www.who.int/docs/default-source/coronaviruse/who-china-joint-mission-on-covid-19-final-report.pdf. Accessed on March 10, 2020. 2020.

15 He X, Lau EHY, Wu P, Deng X, Wang J, Hao X, et al. Temporal dynamics in viral shedding and transmissibility of COVID-19. Nat Med. 2020;26:672-5.

16 Salje H, Tran Kiem C, Lefrancq N, Courtejoie N, Bosetti P, Paireau J, et al. Estimating the burden of SARS-CoV-2 in France. Science. 2020:eabc3517.
17 Heffernan JM, Smith RJ, Wahl LM. Perspectives on the basic reproductive ratio. Journal of the Royal Society Interface. 2005;2:281-93.
